# A Database-Derived Phthalate Ester–Ankylosing Spondylitis Signature Identifies an AP-1/CXCL8 Inflammatory Classical-Monocyte Program

**DOI:** 10.3390/genes17091113

**Published:** 2026-09-14

**Authors:** Xiqing Luo, Xuqi Zheng, Wenyu Xu, Dan Guo, Xinlei Jia, Jieruo Gu, Xiaoyi Zhao, Yutong Jiang

**Affiliations:** 1 Department of Rheumatology and Immunology, Third Affiliated Hospital of Sun Yat-sen University, Guangzhou 510630, China; xiqingluo@outlook.com (X.L.);; 2 Department of Joint Surgery, First Affiliated Hospital of Sun Yat-sen University, Guangzhou 510080, China

**Keywords:** ankylosing spondylitis, phthalate esters, transcriptomic signature, classical monocytes, CXCL8, AP-1, single-cell RNA sequencing

## Abstract

**Objectives:** We tested whether a database-derived phthalate ester (PAE)–ankylosing spondylitis (AS) candidate panel identifies an inflammatory transcriptional program and characterized its transcription-factor (TF) architecture. **Methods:** Machine learning prioritization and repeated nested cross-validation were followed by locked-model transfer from GSE73754 to GSE25101. Donor-level single-cell analyses included 96,746 peripheral blood mononuclear cells from 10 AS and 29 healthy-control donors (GSE194315), with corroboration assessed in 25 additional baseline AS patients (GSE277117). **Results:** Within classical monocytes, the continuous signature covaried with panel-excluded TNF-α/NF-κB signaling (ρ = 0.571; q = 0.000729), inflammatory response (ρ = 0.447; q = 0.0108), and eight of 10 AP-1-related TF activities. Target-excluded TF activities covaried with CXCL8 (8/10) and IL1B (10/10). AP-1-gene/CXCL8 co-expression received partial corroboration in the additional AS cohort (JUN–CXCL8: ρ = 0.92). Among 16 genes prioritized from 473 shared candidates, CXCL8, IL2RB, STAT5B and TNF were stable. The locked 14-gene model achieved an area under the receiver-operating-characteristic curve of 0.770 (DeLong 95% confidence interval, 0.596–0.943), supporting partial rank transportability. Secondary analyses showed a lower CD56bright fraction among natural killer (NK) cells in AS (−2.699 percentage points; 95% confidence interval, −4.575 to −0.611; q = 0.0498) and reduced NK-cell JUN expression (log_2_ fold change = −0.995; adjusted *p* = 0.0497). **Conclusions:** The PAE–AS signature identifies an AP-1/CXCL8-associated classical-monocyte inflammatory program, with partial cross-cohort corroboration and secondary NK alterations, providing candidates for exposure-informed mechanistic studies.

## 1. Introduction

Ankylosing spondylitis (AS) is a chronic immune-mediated inflammatory disease characterized by axial inflammation, extra-articular manifestations and progressive structural damage [1]. Innate immune activation and TNF- and IL-17-associated inflammatory pathways are established components of disease pathogenesis [2], and AS has a strong genetic basis [3]. However, the circulating immune-cell programs associated with inflammatory heterogeneity in AS remain incompletely understood.

Phthalate esters (PAEs) are synthetic chemicals used as plasticizers and other additives in consumer products. Experimental studies indicate that several PAEs and their metabolites can alter innate immune responses in monocyte/macrophage systems. In differentiated THP-1 macrophage-like cells, di-(2-ethylhexyl) phthalate (DEHP) increases the production of inflammatory mediators, including IL-8/CXCL8, and promotes nuclear translocation of NF-κB p65 [4]. In lipopolysaccharide (LPS)-stimulated human peripheral blood mononuclear cell cultures, diethyl phthalate(DEP), di-n-butyl phthalate(DBP) and mono-n-butyl phthalate alter cytokine secretion, with responses differing across cytokines [5]. Maternal butyl benzyl phthalate exposure from before mating through weaning also increases the susceptibility to and severity of collagen-induced arthritis in offspring mice [6]. These findings provide a rationale for examining PAE-related immune programs in immune-mediated disease.

Despite these experimental observations, it remains unclear whether genes linked to PAEs in toxicogenomic and target-prediction resources converge on AS-associated inflammatory programs in specific circulating immune-cell populations. Monocytes are plausible candidates because they integrate innate sensing and cytokine amplification [7,8,9], and single-cell studies have identified disease-associated myeloid-cell alterations in AS, including monocyte abnormalities [10,11,12].

We integrated public bulk-transcriptomic datasets with single-cell profiles of peripheral blood mononuclear cells to examine whether a database-derived PAE–AS overlap signature is associated with circulating inflammatory states. We assessed cross-cohort consistency and cellular localization, with exploratory donor-level analyses of inflammatory and regulatory programs in cell populations prioritized during analysis.

## 2. Materials and Methods

### 2.1. Study Cohorts and Data Sources

Publicly available datasets GSE73754, GSE25101, GSE299639, GSE194315, and GSE277117 were used for bulk and single-cell transcriptomic analyses. None of these cohorts included direct PAE or metabolite measurements. The term PAE–AS signature therefore denotes a database-derived transcriptional construct, not an exposure biomarker. Detailed dataset characteristics and analytical roles are provided in Supplementary Table S1.

### 2.2. Construction of the Database-Derived PAE–AS Candidate Set

Seven common phthalate esters (PAEs), namely dimethyl phthalate (DMP), diethyl phthalate (DEP), diisobutyl phthalate (DiBP), di-n-butyl phthalate (DBP), di(2-ethylhexyl) phthalate (DEHP), diisononyl phthalate (DiNP) and di-n-octyl phthalate (DnOP), were included in this study [13] (Supplementary Table S2). The chemical structures and Simplified Molecular Input Line Entry System (SMILES) strings of these compounds were obtained from PubChem. Potential human targets of PAEs were identified using the Comparative Toxicogenomics Database (CTD) [14], PharmMapper [15] and SwissTargetPrediction [16], with the species restricted to Homo sapiens. The targets obtained from the three databases were integrated, and duplicate entries were removed before subsequent analyses. To comprehensively identify potential targets associated with AS, this study conducted systematic searches across publicly accessible databases: GeneCards (https://www.genecards.org, accessed on 10 August 2026) and Online Mendelian Inheritance in Man (OMIM, https://omim.org, accessed on 10 August 2026). The two target lists were intersected to define the shared PAE–AS candidate pool. Over-representation analysis of Gene Ontology and KEGG terms was performed with gseapy [17], with Benjamini–Hochberg (BH)-adjusted *p* < 0.05 deemed significant.

### 2.3. Machine Learning Prioritization, Stability Assessment and Locked-Model Transfer

The GSE73754 dataset included 72 samples (52 AS and 20 HC) with expression profiles for 27,410 genes. Candidate genes were identified using an intersection strategy: AS-related genes ∩ (CTD ∪ PharmMapper ∪ SwissTargetPrediction-derived target genes). This procedure yielded 473 candidate genes, of which 444 were matched to the GSE73754 expression matrix and retained for downstream analysis. Samples were randomly divided into training and validation sets using stratified sampling to preserve the AS/HC distribution. With a validation proportion of 30% and a fixed random seed of 42, 50 samples (36 AS and 14 HC) were assigned to the training set and 22 samples (16 AS and 6 HC) to the validation set. Missing expression values were imputed using the median. To identify candidate features, gene-importance rankings were further extracted from three representative single models: random forest, LASSO and XGBoost. Consensus genes were defined as genes ranked within the top 20 by at least two of the three algorithms, resulting in 16 machine learning consensus genes. The consensus genes were carried into two additional cohorts for cross-cohort candidate prioritization—a within-cohort refit and cross-validation in each dataset, not a locked-model external validation. In GSE25101, 32 samples (16 AS and 16 HC) were included; 14 of the 16 consensus genes were detected, with *ADAMTS4* and *OLFML3* absent from the expression matrix. In GSE299639, 12 samples (6 AS and 6 HC) were analyzed; 13 consensus genes were detected, with *ADAMTS4*, *OLFML3* and *ALPP* absent. Single-gene separability was assessed by ROC analysis in each cohort. For combined evaluation, logistic regression models based on the matched consensus genes were constructed using median imputation and standardization, and performance was estimated by 5-fold stratified cross-validation within each cohort. Genes with single-gene AUC > 0.5 in both GSE25101 and GSE299639 were retained as cross-cohort-detectable candidate genes.

Feature stability was additionally assessed by 100 repetitions of stratified five-fold outer cross-validation with stratified five-fold inner tuning. A retrospective locked-model analysis then trained a 14-gene L1-penalized logistic regression model in GSE73754 and applied it unchanged to GSE25101.

### 2.4. Single-Cell RNA-Sequencing Processing and Monocyte Annotation

The public single-cell RNA-sequencing dataset GSE194315 included 10 ankylosing spondylitis (AS) samples and 29 healthy controls (HCs). Data processing, integration, clustering and visualization were performed in Python using Scanpy and OmicVerse v1.7.9. After quality control, cells with a mitochondrial gene percentage greater than 10% or with low detected feature counts were excluded. The remaining cells were subjected to normalization, highly variable gene selection, scaling, principal component analysis, Harmony-based batch correction [18], neighborhood graph construction, UMAP embedding and Leiden clustering. Major PBMC lineages were annotated by integrating canonical marker-based inspection, curated marker-set scoring, cluster-level enrichment patterns and reference-based prediction using CellTypist on the normalized full-gene expression matrix. For monocyte-focused analysis, cells assigned to the monocyte lineage were extracted and reprocessed independently. Monocytes were re-clustered on canonical markers (CD14, FCGR3A, MS4A7) into classical (CD14^++^CD16^−^), intermediate (CD14^+^CD16^+^) and non-classical (CD14^−^CD16^++^) subsets. For each monocyte, the mean log-normalized expression of the 16 consensus genes defined the PAE–AS overlap score. For visualization, cells were partitioned into PAE–AS signature-low/PAE–AS signature-mid/PAE–AS signature-high by tertile (pandas qcut, q = 3); these were operational groups, not measured exposure strata or biological thresholds. Hallmark module activity was quantified per cell with AUCell [19,20].

### 2.5. Exploratory Cell-Level Differential Expression and Gene-Set Enrichment

Exploratory cell-level differential expression was computed for classical monocytes by signature-defined group (PAE–AS signature-high vs. PAE–AS signature-low) and by group (AS vs. HC); the resulting DEGs were ranked by log_2_ fold-change and adjusted *p* value and submitted to gseapy [17] against the MSigDB Hallmark collection [20], with BH-adjusted *p* < 0.05 considered significant. Because per-cell tests treat individual cells as independent replicates and thereby inflate significance (pseudoreplication), all inferential single-cell comparisons in this study were re-derived at the donor level; cell-pooled statistics results are retained in Supplementary Table S3 solely as exploratory context.

### 2.6. Donor-Level Pseudobulk Differential-Expression Analysis

To obtain donor-level statistical inference across the 39 biological replicates, raw UMI counts from the full-gene matrix (16,897 genes) were aggregated per donor × cell type × signature group. Only donor-compartment pseudobulk profiles built from ≥20 cells (NK) or ≥10 cells (classical-monocyte tertile bins) were retained. Two contrasts were run. (i) NK cells, AS vs. HC (10 AS, 29 HC): Summed counts were modeled with pydeseq2/DESeq2 (design ~ group; genome-wide BH-adjusted *p*), complemented by per-donor Mann–Whitney U tests on log-CPM for a targeted effector-gene panel. (ii) Classical monocytes, PAE–AS signature-high vs. PAE–AS signature-low: Because both tertiles are sampled within each donor, a within-donor paired design was used—per-donor log-CPM values for the two tertiles were compared by paired Wilcoxon signed-rank tests across donors contributing both tertiles (n = 39 tertile-pairs). Age and sex were available for all 39 donors, whereas treatment, disease duration, BASDAI, ASDAS, CRP and ESR were incomplete or unavailable. Donor identity was handled through donor-level aggregation and within-donor pairing; monocyte sensitivity models were additionally adjusted for age, sex and donor-level technical-batch fractions. In the primary continuous analyses, counts were also summed per donor and cell type (≥20 cells) and transformed to log-CPM; available signature genes were standardized across donors before their unweighted mean was calculated. Score differences were summarized by mean differences and Hedges’ g, with 5000 donor-bootstrap confidence intervals, Mann–Whitney tests and BH correction across the tested cell types. For classical-monocyte pathway associations, the 16 signature genes were excluded from the tested Hallmark scores and transcriptome-wide ranking; the continuous-score count model used design ~ group + signature_score. BH correction was applied genome-wide (DESeq2) or across the targeted gene panel, as stated per analysis.

### 2.7. Donor-Level Immune-Cell Composition Analysis

To distinguish compositional shifts from within-subset reprogramming, donor-level endpoints were total NK cells among PBMCs, CD56bright cells among NK cells and the author-provided generic NK category among NK cells. The generic category was termed conventional NK and was not relabeled CD56dim. Group differences were assessed by ordinary least-squares regression of arcsine-square-root-transformed proportions with BH correction across the three endpoints. The composition analysis included 10,064 NK-compartment cells from 39 donors (10 AS and 29 HC); within-NK endpoints required at least 20 NK cells per donor. Mean differences in percentage points and 95% confidence intervals were summarized using 5000 donor-bootstrap resamples.

### 2.8. Additional-Cohort Analysis of AP-1/CXCL8 Covariation

Reproducibility of the monocyte AP-1-gene/CXCL8 covariation was assessed in an additional AS scRNA-seq cohort (GSE277117), a TNF/IL-17-inhibitor treatment-response study containing pre- and post-treatment PBMC samples. Only baseline (“base”) libraries from genuine single patients were retained; genotype-demultiplexed multiplet cells were excluded, and where a patient contributed more than one baseline treatment arm, the arms were aggregated to the patient. Cells were normalized and Leiden-clustered; monocyte clusters were identified by mean monocyte-marker score (LYZ, CD14, FCN1, S100A8, S100A9, VCAN). Patient-level pseudobulk mean expression was computed for patients with ≥20 monocytes, and Spearman correlations between AP-1 gene expression (JUN, JUNB, FOS, JUND, EGR1) and ligand expression (CXCL8, IL1B, TNF, HLA-E) were evaluated across patients. The additional-cohort correlation analysis assessed gene-expression covariation rather than ULM-inferred TF activity. Separate patient-level sensitivity analyses assessed full-signature–TF associations and target-excluded TF–CXCL8 associations. Because this cohort lacked healthy controls, this analysis can only partially corroborate AP-1-gene/CXCL8 covariation and cannot replicate the AS-versus-HC contrasts.

### 2.9. Transcription-Factor Regulon and Activity Analysis

TF regulatory networks were reconstructed using pySCENIC [21] applied to classical monocytes (n = 12,014). Adjacencies were inferred from the expression matrix (31,310 TF–target edges), and regulon specificity scores (RSS; 1—Jensen–Shannon divergence) were computed per cell group to quantify regulon specificity across signature groups and disease conditions. TF activity in individual cells was independently estimated using the decoupler univariate linear model (ULM) [22] with the CollecTRI TF–target interaction database [23]. For each AP-1 family member (JUN, JUND, JUNB, FOS, FOSB, ATF3) and related TFs (EGR1, KLF4, KLF6, NR4A2), ULM activity was inferred and compared across signature groups. To avoid pseudoreplication, the descriptive paired-tertile AP-1 comparison was performed at the donor level: classical-monocyte counts were pseudobulked per donor × signature tertile, ULM activity was recomputed on these donor-tertile profiles, and PAE–AS signature-high versus PAE–AS signature-low activity was compared by within-donor paired Wilcoxon signed-rank tests across donors contributing both tertiles, with Benjamini–Hochberg correction across the 10 TFs; cell-level Mann–Whitney comparisons are reported only descriptively. TF activity was also compared between AS and HC at the donor level (per-donor classical-monocyte pseudobulk ULM activity, Mann–Whitney U with BH correction across the 10 TFs); because no TF was disease-associated after correction, the module is not sub-classified into disease-responsive subsets. Primary continuous analyses related continuous donor-level signature scores to TF activity; removed each tested ligand from its corresponding regulon before recalculation; and adjusted for age, sex and technical-batch fractions.

### 2.10. Donor-Level Transcription-Factor–Ligand Covariation

Donor-aggregated classical-monocyte profiles were generated by summing raw UMI counts per donor (≥20 cells; n = 39 donors) and transforming them to log-CPM. ULM TF activity was recomputed on these donor-level profiles after each tested ligand was excluded from the regulatory network where present. Spearman correlations were computed between each AP-1 TF activity and the expression of CXCL8, IL1B, TNF and HLA-E to assess covariation with the selected inflammatory genes, with HLA-E included as a reference gene. Confidence intervals were calculated using 5000 donor-bootstrap resamples, and BH correction was applied across the 10 TFs separately for each ligand.

### 2.11. Donor-Level AP-1 Divergence Analysis in NK Cells

To quantify relative inferred JUN and JUNB activity, a per-cell AP-1 divergence score was defined as Z(JUN) − Z(JUNB), where Z denotes the z-score-normalized ULM activity. Positive values indicate JUN-dominant, and negative values indicate JUNB-dominant AP-1 composition. For the donor-level AP-1 divergence analysis, scores were averaged within each donor and compared between AS and HC using a Mann–Whitney U test (29 HC and 10 AS donors). JUN and JUNB regulon target genes were obtained from the CollecTRI database to identify exclusive versus shared effector gene targets.

### 2.12. Statistical Analysis and Software

Analyses were run using Python 3.10 (omicverse 1.7.9, scanpy 1.11.3, decoupler 1.7.0, pySCENIC 0.12.1). Unless otherwise stated, between-group comparisons used Mann–Whitney U tests with BH correction; adjusted *p* < 0.05 defined significance.

## 3. Results

### 3.1. Database Intersection Yields 473 Inflammation-Enriched Candidate Genes

**Figure 1:**
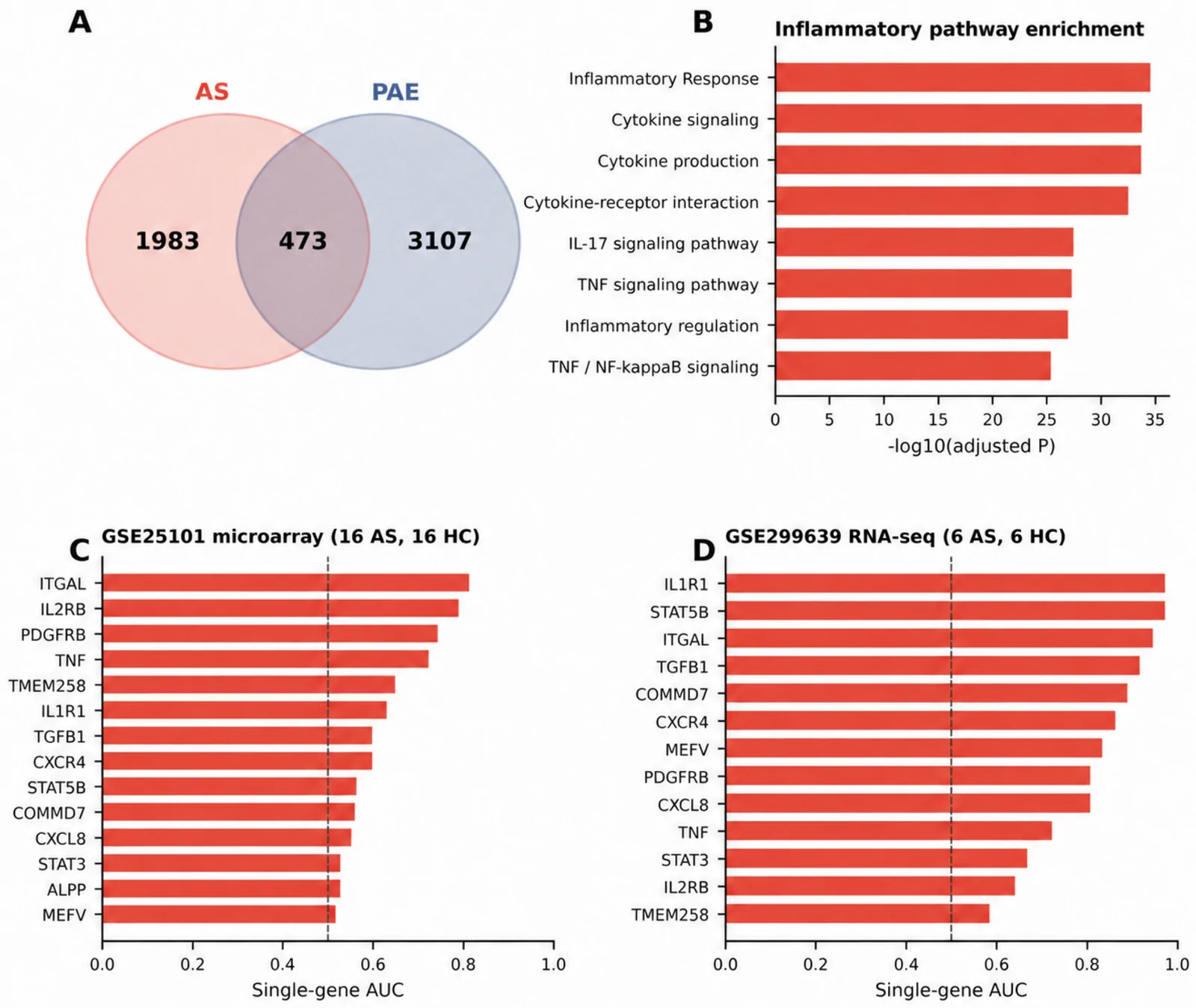
A database-derived PAE–AS overlap inflammatory gene program. (**A**) Venn intersection of PAE-target and AS-associated genes (473 shared). (**B**) Over-representation of the intersection across NF-κB/TNF/IL-17 inflammatory pathways. (**C**) GSE25101 microarray detection and single-gene separability. (**D**) GSE299639 RNA-seq detection and single-gene separability.

Figure S1 shows the detailed workflow of the proposed method. The archived AS-associated set contained 2456 genes, and the union of genes associated with the seven PAEs contained 3580 genes. Integrating database-derived PAE-associated or predicted targets with AS-associated genes returned 473 shared candidate genes (Figure 1A). Functional enrichment of the intersection was dominated by inflammatory response, cytokine–cytokine receptor interaction, NF-κB signaling, TNF signaling and IL-17 signaling (Figure 1B), showing overlap with broad NF-κB/TNF/IL-17 inflammatory circuitry but not establishing PAE specificity.

### 3.2. Four Genes Are Stable, and Locked-Model Transferability Is Limited

In the GSE73754 training cohort, 444 candidate genes were analyzed using the LASSO, random forest (RF) and XGBoost algorithms. Intersecting the top-ranked features across at least two methods yielded a consensus panel of 16 genes: ADAMTS4, STAT5B, COMMD7, CXCR4, TGFB1, OLFML3, MEFV, IL2RB, IL1R1, TMEM258, ALPP, TNF, CXCL8, ITGAL, PDGFRB and STAT3 (Table 1). Each algorithm generated 500 held-out outer-fold fits. Cross-algorithm consensus selection classified four of the 16 panel genes as stable: CXCL8 (selection frequency, 0.722), IL2RB (0.852), STAT5B (0.994), and TNF (0.740). Five genes were classified as intermediate: ADAMTS4 (0.500), COMMD7 (0.506), MEFV (0.368), OLFML3 (0.360), and TGFB1 (0.546). The remaining seven genes had consensus frequencies below 0.30. In particular, IL1R1, ITGAL, and PDGFRB were each selected in no more than 0.004 of outer fits (Table 1). Of these, 13 were detectable in both additional cohorts (Figure 1C,D; Table 1). We used this panel for candidate-gene prioritization; in each additional cohort, a logistic regression model was refit and evaluated by internal 5-fold cross-validation (AUC 0.66 in GSE25101; 0.97 in GSE299639; Figure S2).

Applied unchanged to GSE25101, the locked 14-gene LASSO yielded an AUC of 0.770 (DeLong 95% CI, 0.596–0.943). At the frozen threshold of 0.443, sensitivity was 0.562 and specificity 0.812; the Brier score was 0.241 and the calibration slope 0.443.

### 3.3. Donor-Level Scores Do Not Preferentially Localize to Classical Monocytes

**Figure 2:**
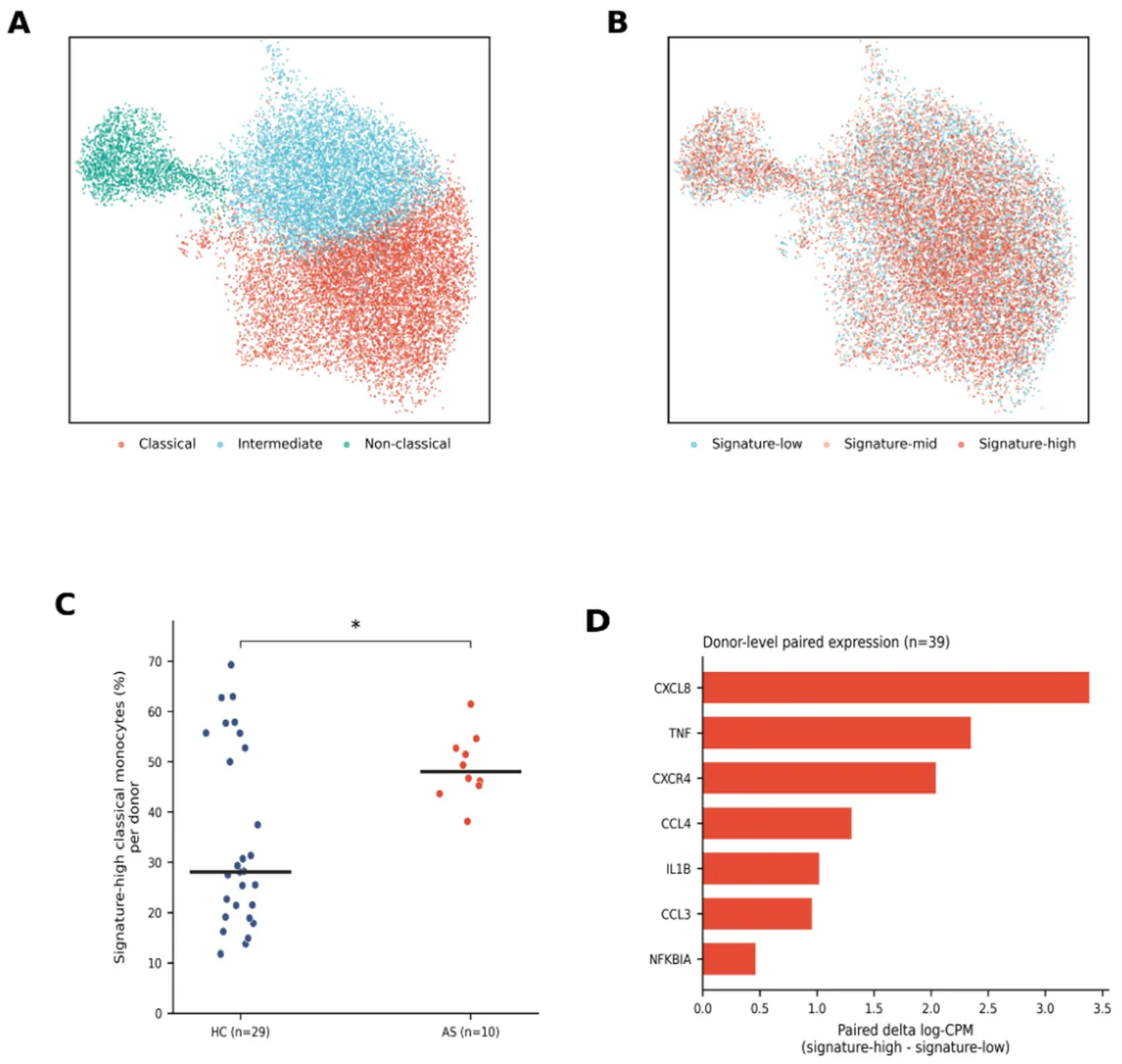
Operational PAE–AS signature groups and descriptive donor-level classical-monocyte contrasts. (**A**) UMAP of re-clustered monocytes colored by monocyte subtype. (**B**) The same UMAP colored by operational PAE–AS signature group. (**C**) Percentage of signature-high classical monocytes per donor (29 HC and 10 AS donors); the unadjusted tertile-based AS–HC comparison was nominally different (48% vs. 28%, *p* = 0.038; * denotes *p* < 0.05), but the corrected continuous-score analysis was nonsignificant. (**D**) Donor-level paired expression in signature-high versus signature-low classical monocytes (descriptive score-defined contrast; Supplementary Table S4). Panels (**C**,**D**) are descriptive; panel (**D**) includes genes that contribute to the score and is not an independent validation of the signature.

From GSE194315 (10 AS and 29 HC), the PBMC atlas resolved major immune lineages, and re-clustering recovered 23,401 monocytes across classical, intermediate and non-classical subsets (Figure 2A,B). No major lineage or monocyte subset showed a significant AS–HC continuous-score difference after BH correction. In CD14 classical monocytes, the AS–HC mean difference was 0.096 standardized units and Hedges’ g was 0.558 (95% CI, −0.208 to 1.407; *p* = 0.182; q = 0.364). The frozen tertile display showed a larger signature-high fraction in AS (48% vs. 28%; *p* = 0.038), but tertiles were operational, and the corrected continuous analysis was nonsignificant.

### 3.4. The Classical-Monocyte Signature Covaries with Inflammatory Pathways

Because panel genes contribute to the PAE–AS overlap score, the primary donor-level analysis excluded panel genes from the transcriptome-wide ranking and from the tested Hallmark programs. The continuous CD14-monocyte score correlated with TNF-α/NF-κB (ρ = 0.571; q = 0.000729) and inflammatory response (ρ = 0.447; q = 0.0108), and both associations persisted after disease-group adjustment. In the donor-level model, 1085 genes were associated with the score after BH correction, falling to 997 after age/sex adjustment and 104 after additional technical-batch adjustment. These results support a coordinated within-cohort inflammatory pattern but do not establish batch independence or PAE specificity.

### 3.5. The Signature Covaries with an Inferred AP-1/Immediate-Early Transcription-Factor Module

**Figure 3:**
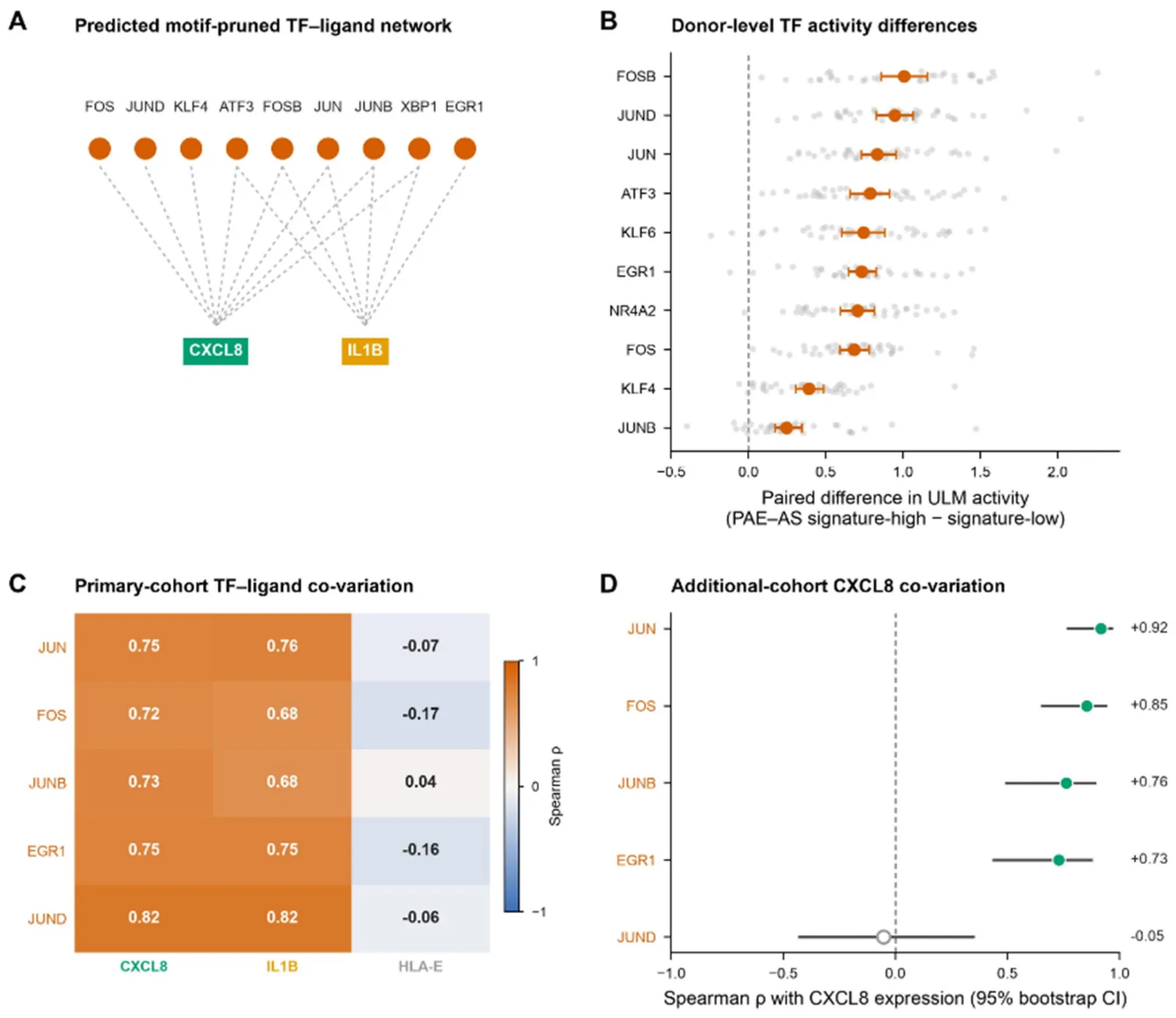
An inferred AP-1/immediate-early transcriptional module covaries with CXCL8/IL1B, with concordant CXCL8 covariation in an additional AS cohort. (**A**) Motif-pruned pySCENIC regulon network displaying nine TFs with retained predicted edges to CXCL8 and/or IL1B. The initial adjacency-stage 10-TF ranking is described separately in Results. Dashed edges denote computationally inferred TF–target relationships and do not establish causal regulation. (**B**) Donor-level paired differences in decoupler ULM TF activity between signature-high and signature-low classical monocytes from 39 donors. Gray points show donor-level paired differences; orange points and horizontal lines indicate Hodges–Lehmann paired-difference estimates and 95% donor-bootstrap confidence intervals. Effect estimates are signature-high minus signature-low. This paired-tertile display is descriptive; primary inference used continuous donor-level signature associations. (**C**) Donor-level Spearman correlations between selected TF activities and CXCL8, IL1B and HLA-E across the primary cohort (n = 39 donors). Each tested ligand was excluded from its corresponding regulon, where present, before ULM activity was recalculated; the heatmap shows the unadjusted donor-level correlations. HLA-E is shown as a reference gene with minimal covariation. The five TFs shown in panels (**C**,**D**) were selected for a focused cross-cohort display and are presented in the same order. (**D**) Patient-level gene-expression covariation in an additional AS cohort (GSE277117; n = 25 baseline patients). Points and horizontal lines indicate Spearman correlations with CXCL8 and 95% bootstrap confidence intervals. These associations represent transcriptional covariation rather than TF-activity measurements or causal regulatory effects.

To characterize TF architecture associated with CXCL8 and IL1B co-expression, we applied pySCENIC to classical monocytes. At the initial adjacency stage (31,310 edges), CXCL8 and IL1B shared the same 10 top-ranked candidate TFs, an AP-1-centered immediate-early/stress-response module (JUN, JUNB, JUND, FOS, FOSB, ATF3, EGR1, KLF4, KLF6 and NR4A2): EGR1 (importance 12.1), FOS (11.4), JUN (10.3), FOSB (9.1), KLF6 (8.6), KLF4 (8.2), JUND (7.9), JUNB (6.9), ATF3 (5.6) and NR4A2 (5.4). After motif pruning, the displayed regulon network retained nine TF nodes and target-specific predicted edges to CXCL8 and/or IL1B (Figure 3A); these computational relationships do not establish causal regulation. The same adjacency-stage module also ranked among the top candidate regulators of NFKBIA, with GRN importance scores reported for candidate ranking only. The 10/10 adjacency-stage overlap across CXCL8, IL1B and NFKBIA suggests a shared inferred module associated with inflammatory gene expression; it does not establish relative regulatory strength or a link to IFN-γ signaling.

Historical RSS analysis descriptively suggested higher AP-1 regulon specificity in signature-high than signature-low classical monocytes. In the primary donor-level continuous analysis, eight of 10 prespecified TF activities were associated with the signature after BH correction: JUN, JUND, JUNB, FOS, FOSB, ATF3, EGR1 and KLF6; JUND had the strongest association (ρ = 0.576; q = 0.00127). Separately, the archived paired signature-high versus signature-low comparison showed positive Hodges–Lehmann differences for all 10 TFs (Figure 3B; Supplementary Table S5). None of the 10 TF activities differed significantly between AS and HC.

### 3.6. AP-1/CXCL8 Covariation Receives Partial Corroboration in an Additional AS Cohort

To assess AP-1–cytokine covariation at the individual level, we generated donor-aggregated monocyte profiles from 39 donors (10 AS and 29 HC). After each ligand was excluded from its corresponding regulons where present, eight of 10 TF activities covaried with CXCL8, 10 of 10 with IL1B, seven of 10 with TNF, and none with HLA-E (selected TF–ligand pairs are shown in Figure 3C). Six CXCL8 and seven IL1B associations remained after age/sex/batch adjustment, whereas none of the TNF associations did; no target-excluded association survived correction within the 10 AS donors. In GSE277117, 25 baseline AS patients were retained, but the cohort had no HC. Six full-signature–TF and six target-excluded TF–CXCL8 associations survived correction, although the contributing TFs and directions were not identical; in the separate gene-expression analysis, JUN–CXCL8 was strong (ρ = 0.92), whereas JUND–CXCL8 was weak (ρ = −0.05; Figure 3D). This is partial within-AS corroboration, not replication of an AS-specific regulatory program or direct TF regulation.

### 3.7. NK-Cell Findings Are Secondary and Primarily Compositional

**Figure 4:**
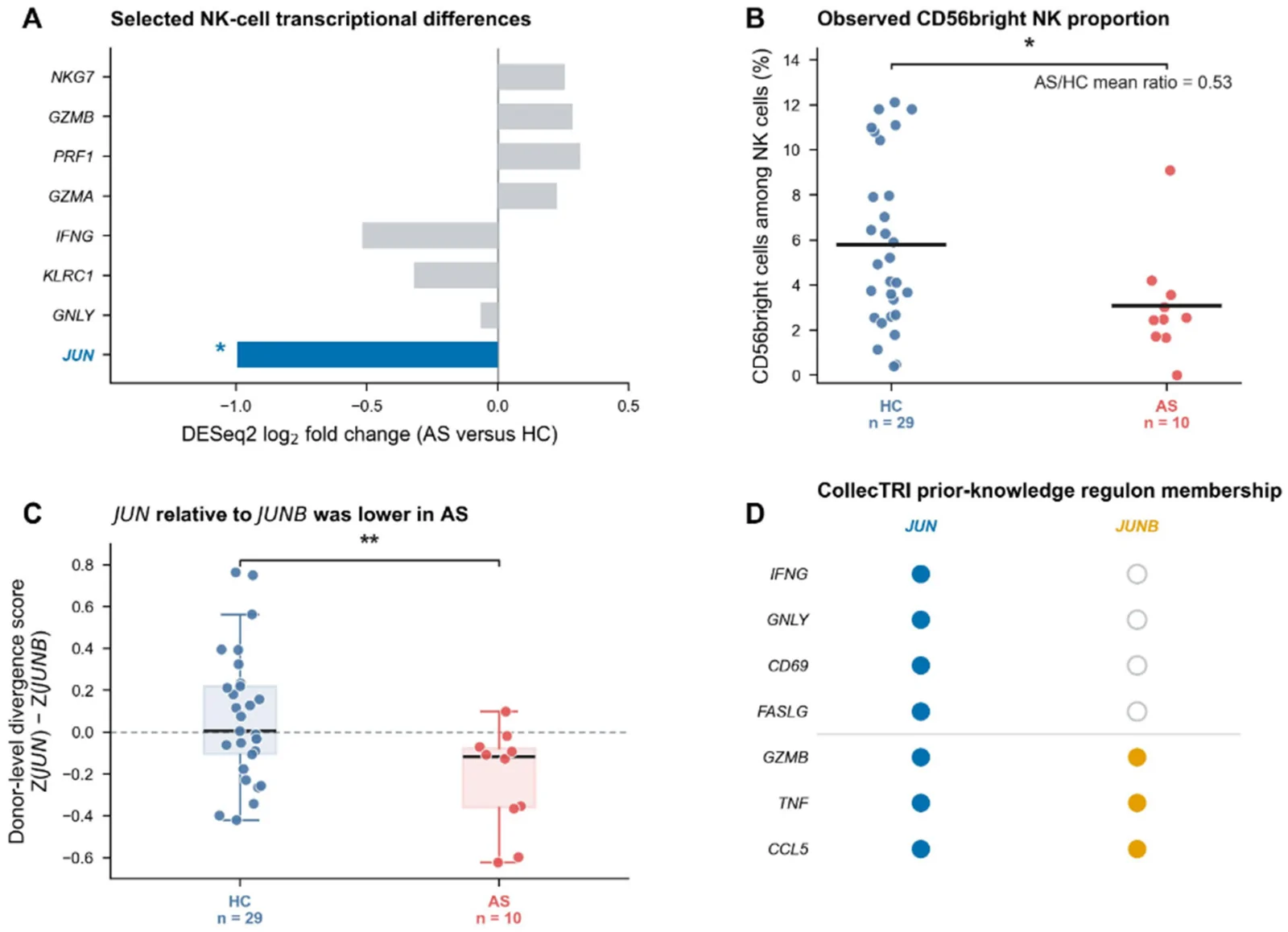
Secondary donor-level NK-cell findings and AP-1 regulon context. (**A**) Selected AP-1 factors and NK effector genes from donor-level NK-cell pseudobulk DESeq2 comparing AS with HC (10 AS and 29 HC donors). JUN showed log_2_FC = −0.995 (95% CI, −1.553 to −0.437; *p* = 4.77 × 10^−4^; BH-adjusted *p* = 0.0497); the remaining selected genes were nonsignificant (adjusted *p* ≥ 0.17). (**B**) Observed CD56bright NK-cell proportions per donor (29 HC and 10 AS donors); the descriptive AS/HC mean ratio was 0.53. The AS–HC mean difference was −2.699 percentage points (95% donor-bootstrap CI, −4.575 to −0.611); the prespecified arcsine-square-root OLS model yielded *p* = 0.0332 and BH q = 0.0498. (**C**) Donor-level AP-1 divergence score, Z(JUN) − Z(JUNB), based on standardized inferred ULM activities and averaged per donor, was lower in AS (*p* = 9.6 × 10^−3^; 29 HC and 10 AS donors). (**D**) CollecTRI prior-knowledge regulon membership: IFNG, GNLY, CD69 and FASLG were annotated as targets of JUN but not JUNB in this database, whereas GZMB, TNF and CCL5 were shared by JUN and JUNB. In panels (**A**,**B**), * denotes BH-adjusted *p* < 0.05; in panel (**C**), ** denotes descriptive *p* < 0.01.

We characterized the NK compartment at the donor level (pseudobulk, 10 AS vs. 29 HC; DESeq2), the appropriate unit given 39 biological replicates. The “effector dissociation” impression from cell-pooled testing—increased cytotoxic transcripts (PRF1, GZMB, NKG7) with decreased IFNG/GNLY—did not survive donor-level analysis: none of these effector genes reached significance after Benjamini–Hochberg correction (PRF1 padj = 0.23, GZMB 0.27, NKG7 0.17, IFNG 0.58, and GNLY 0.92; all padj ≥ 0.17; Figure 4A; Supplementary Table S6). The corresponding cell-pooled *p* values (e.g., PRF1 3.6 × 10^−30^, GZMB 9.1 × 10^−28^) reflect pseudoreplication across thousands of cells rather than 39 independent donors. Among the selected NK genes, JUN reached donor-level significance, with reduced expression in AS (DESeq2 log_2_FC = −0.995, 95% CI −1.553 to −0.437, *p* = 4.77 × 10^−4^, exact padj = 0.0497); this transcript-level association does not establish a functional NK-cell program.

Donor-level compositional analysis identified differences in NK-subset proportions. AS donors had a lower CD56bright fraction (−2.699 percentage points; 95% CI, −4.575 to −0.611; q = 0.0498) and a higher conventional-NK fraction (+3.035 percentage points; 95% CI, 1.062–4.839; q = 0.0498) (Figure 4B; Supplementary Table S7). The total NK fraction among PBMCs did not differ significantly (−0.230 percentage points; 95% CI, −4.030 to 4.011; q = 0.9677; Supplementary Table S7). Differences in NK-subset composition may contribute to the pooled IFNG pattern; these analyses do not establish reduced IFN-γ output or cell-intrinsic reprogramming.

Consistent with the donor-level JUN reduction, a per-patient AP-1 divergence score (Z_JUN − Z_JUNB, averaged per donor to avoid pseudoreplication) shifted from mildly JUN-dominant in HC (mean +0.071) to JUNB-dominant in AS (mean −0.226; Mann–Whitney *p* = 9.6 × 10^−3^, n = 39) (Figure 4C). In the CollecTRI reference network, IFNG, GNLY, CD69 and FASLG are annotated as targets of JUN but not JUNB, whereas GZMB, TNF and CCL5 are shared JUN/JUNB targets (Figure 4D). These database annotations provide regulatory context but do not establish JUN-specific functional target programs in AS NK cells.

## 4. Discussion

This study associates a database-derived PAE–AS signature with inflammatory pathways and inferred AP-1 activity in donor-level classical-monocyte profiles. AP-1-related activity covaried with CXCL8 and IL1B, and AP-1-gene/CXCL8 covariation received partial within-AS corroboration in an additional AS cohort without HC. The corrected continuous signature score and the tested TF activities did not differ significantly between AS and HC, so these associations do not establish an AS-specific program. In the NK compartment, donor-level analysis identified a lower relative abundance of the CD56bright subset and reduced JUN expression. These NK observations provide secondary cellular context.

Experimental studies of individual PAEs provide biological context for the observed transcriptional overlap. DEHP altered inflammatory signaling in differentiated THP-1 macrophage-like cells [4]. Phthalates and related metabolites produced cytokine-dependent responses in human PBMC cultures [5]. These experimental findings do not identify phthalate exposure as the upstream cause of the observed immune states. Micro- and nanoplastic particles are chemically and biologically distinct from PAEs and are not used here as mechanistic evidence. An alternative interpretation is that the overlap signature captures a shared inflammatory program represented in both toxicogenomic annotations and AS-associated immune states.

CXCL8 remained a central observational feature as a stable machine learning-panel component, a score-stratified monocyte DEG, and a transcript that covaried with inferred AP-1 activity. These analyses share expression data, and CXCL8 contributes to the signature score, so their convergence is not independent validation. AP-1 and NF-κB participate in stimulus-induced CXCL8 transcription in human monocytic cells [24]. Clinically, reported associations of circulating IL-8/CXCL8 with AS disease-activity indices (BASDAI and ASDAS) [25] support its disease relevance, although the clinical significance of the monocyte transcriptional state identified here remains to be established. The additional AS-only cohort provided partial corroboration of AP-1-related gene–CXCL8 co-expression but could not replicate AS–HC differences. Gene co-expression, regulon-based TF-activity inference, and motif-supported predicted TF–target relationships are complementary computational observations; they do not measure TF protein activation, direct binding or cytokine secretion. The inferred network also included NFKBIA (IκBα), consistent with inflammatory transcription accompanied by NF-κB feedback regulation. These associations remain observational and do not establish direct AP-1 regulation.

The NK findings warrant a cautious interpretation. Donor-level analysis identified a lower relative CD56bright fraction in the primary model and reduced JUN expression (padj = 0.0497). The apparent “effector dissociation” (retained cytotoxic transcripts with reduced IFNG) was not statistically supported once analyzed per donor. Differences in NK-subset composition may contribute to the pooled IFNG pattern, which did not establish cell-intrinsic functional reprogramming. The per-donor JUN–JUNB inferred-activity contrast suggests heterogeneity among AP-1-related transcriptional changes, without establishing distinct functional target programs. Given the reliance on single-database regulon annotation and the absence of protein-level or functional data, these observations provide secondary cellular context and require confirmation in larger cohorts.

Several limitations should temper interpretation. The PAE–AS overlap gene set is computationally inferred rather than derived from measured phthalate-exposure responses, so its specificity for PAEs over general inflammatory activation remains unestablished. No cohort included measurements of PAEs or their metabolites. The sample size for donor-level analyses (n = 39; 10 AS) limits statistical power, and incomplete clinical metadata limited assessment of treatment, disease duration and disease activity as alternative explanations for the observed immune states. Covariate sensitivity analyses of monocyte programs did not eliminate residual confounding, and the cross-sectional design precludes inference about disease initiation versus consequence. PBMC NK phenotypes may differ from tissue-resident NK cells at sites of inflammation. Future work should pair quantified phthalate metabolites with longitudinal single-cell profiling in AS and HC cohorts, record treatment and disease activity, and directly test candidate AP-1/CXCL8 relationships in targeted perturbation experiments with protein-level readouts.

The bulk analyses support candidate-gene prioritization rather than a clinically deployable classifier. Only four of 16 genes met the prespecified stability threshold, and the small evaluation cohorts limit predictive claims. The model trained in GSE73754 and applied unchanged to GSE25101 showed partial rank transportability but limited calibration and frozen-threshold performance. Together, these analyses prioritize an AP-1/CXCL8-associated classical-monocyte state for further investigation in AS; the contribution is the cellular and regulatory characterization of a database-derived overlap signature, rather than evidence of phthalate-induced disease.

## 5. Conclusions

The database-derived PAE–AS signature identifies an AP-1/CXCL8-associated inflammatory program in donor-level CD14 classical-monocyte profiles, with covariation across broad inflammatory pathways and partial within-AS corroboration of AP-1-gene/CXCL8 co-expression. Lower relative CD56bright NK-cell abundance and reduced NK-cell JUN expression provide secondary immune context.

## Figures and Tables

**Table 1:** Sixteen machine learning consensus genes and cross-cohort single-gene separability.

Gene	Method	GSE25101 AUC	GSE299639 AUC	Consensus Selection Frequency	Stability Class
ADAMTS4	LASSO, RF, XGBoost	—	—	0.500	Intermediate
STAT5B	LASSO, RF, XGBoost	0.56	0.97	0.994	Stable
COMMD7	LASSO, RF, XGBoost	0.56	0.89	0.506	Intermediate
CXCR4	LASSO, RF, XGBoost	0.60	0.86	0.140	Unstable
TGFB1	RF, XGBoost	0.60	0.92	0.546	Intermediate
OLFML3	LASSO, RF	—	—	0.360	Intermediate
MEFV	RF, XGBoost	0.52	0.83	0.368	Intermediate
IL2RB	LASSO, RF	0.79	0.64	0.852	Stable
IL1R1	LASSO, XGBoost	0.63	0.97	0.004	Unstable
TMEM258	RF, XGBoost	0.65	0.58	0.178	Unstable
ALPP	RF, XGBoost	0.53	—	0.048	Unstable
TNF	LASSO, RF	0.72	0.72	0.740	Stable
CXCL8	LASSO, XGBoost	0.55	0.81	0.722	Stable
ITGAL	LASSO, RF	0.81	0.94	0.004	Unstable
PDGFRB	LASSO, RF	0.74	0.81	0.004	Unstable
STAT3	RF, XGBoost	0.53	0.67	0.044	Unstable

In GSE25101, ADAMTS4 and OLFML3 lacked represented probes in the analyzed microarray matrix. In GSE299639, ADAMTS4, OLFML3 and ALPP were removed by the prespecified DESeq2-based low-count filter. Consensus selection frequency is the proportion of 500 outer fits in which a gene was selected by at least two algorithms; frequencies of ≥0.60, 0.30–0.59 and <0.30 were classified descriptively as stable, intermediate and unstable. AUC values are single-gene separability estimates; an em dash indicates that the gene was unavailable in that cohort.

## Data Availability

All datasets analyzed in this study are publicly available in the NCBI Gene Expression Omnibus under accessions GSE73754, GSE25101, GSE299639, GSE194315 and GSE277117.

## Supplementary Materials

The following supporting information can be downloaded at https://www.mdpi.com/article/10.3390/genes17091113/s1: Figure S1. Study overview and evidence hierarchy of the PAE–AS signature and monocyte inflammatory context. Figure S2. Secondary within-cohort cross-validated ROC estimates in two additional cohorts. Table S1. Study datasets. Table S2. Chemical information for seven PAEs. Table S3. Exploratory cell-pooled top DEGs, PAE–AS signature-high vs. PAE–AS signature-low classical monocytes (descriptive context only). Table S4. Donor-level classical-monocyte pseudobulk differential expression, PAE–AS signature-high vs. PAE–AS signature-low (within-donor paired Wilcoxon, n = 39 donor tertile-pairs). Table S5. Donor-level paired AP-1/immediate-early transcription-factor activity (decoupler ULM), PAE–AS signature-high vs. PAE–AS signature-low classical monocytes (n = 39 donor tertile-pairs). Table S6. Donor-level NK-cell pseudobulk differential expression, AS vs. HC (DESeq2; 10 AS, 29 HC). Table S7. Donor-level NK-cell composition in AS versus HC.

## Abbreviations

Abbreviation	Definition
AS	ankylosing spondylitis
PAE	phthalate ester
SD	standard deviation
PBMC	peripheral blood mononuclear cell
HC	healthy control
CI	confidence interval
CTD	Comparative Toxicogenomics Database
GRN	gene regulatory network
OMIM	Online Mendelian Inheritance in Man
GO	Gene Ontology
KEGG	Kyoto Encyclopedia of Genes and Genomes
MSigDB	Molecular Signatures Database
ROS	reactive oxygen species
BH	Benjamini–Hochberg
NF-κB	nuclear factor kappa B
MAPK	mitogen-activated protein kinase
LASSO	least absolute shrinkage and selection operator
TNF	tumor necrosis factor
RF	random forest
IL	interleukin
ROC	receiver operating characteristic
IFN-γ	interferon gamma
AUC	area under the curve
DEG	differentially expressed gene
NK	natural killer (cell)
STAT	signal transducer and activator of transcription
UMAP	uniform manifold approximation and projection
BASDAI	Bath Ankylosing Spondylitis Disease Activity Index
ASDAS	Ankylosing Spondylitis Disease Activity Score
AP-1	activator protein-1
TF	transcription factor
ULM	univariate linear model
RSS	regulon specificity score
CollecTRI	comprehensive TF–target interaction database
