# Supplementary material for: A Database-Derived Phthalate Ester–Ankylosing Spondylitis Signature Identifies an AP-1/CXCL8 Inflammatory Classical-Monocyte Program
Source: Genes (Basel). 2026 Sep 14;17(9):1113. doi: 10.3390/genes17091113 (PMC13606070; doi:10.3390/genes17091113)
Supplement: Supplementary file 1 [file genes-17-01113-s001.zip › genes-4526816-supplementary.pdf]

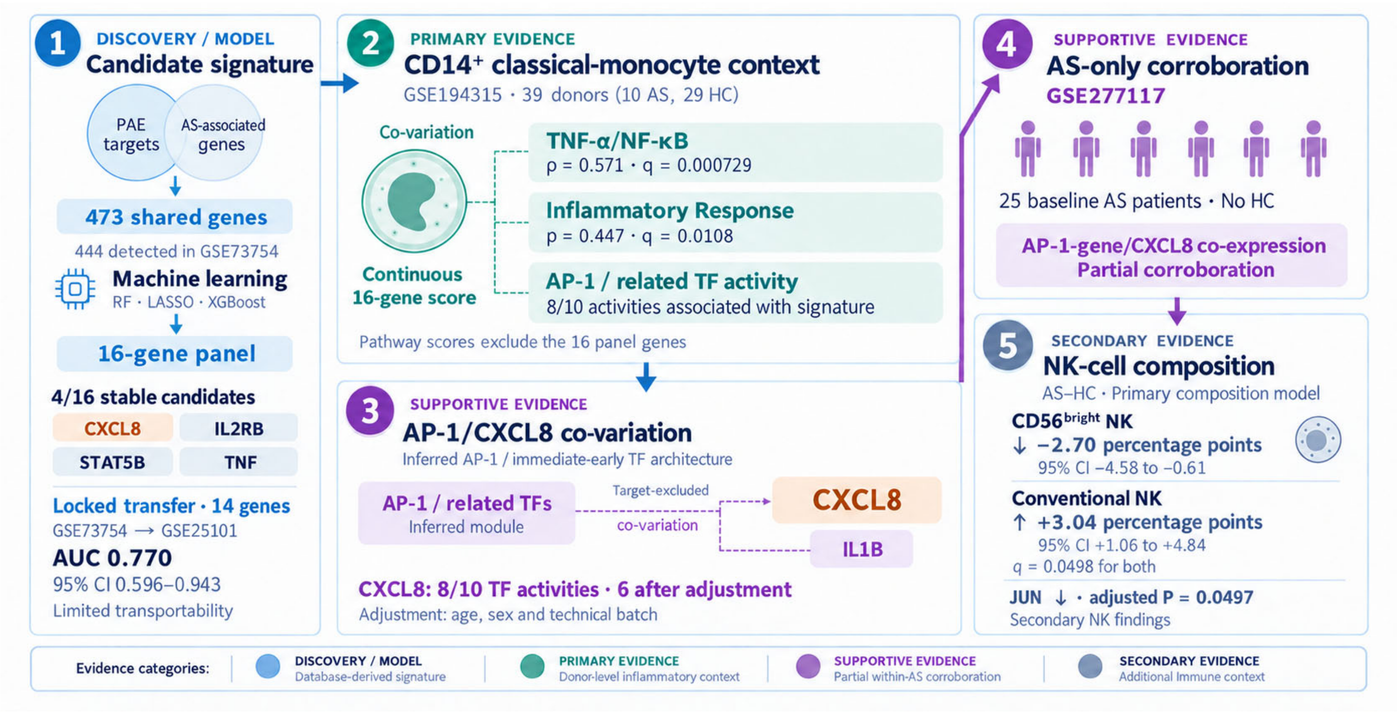

Figure S1. Study overview and evidence hierarchy of the PAE–AS signature and monocyte inflammatory context. Numbered panels indicate the reading order: (1) database-derived candidate construction, machine-learning prioritization, feature-stability assessment and locked-model transfer; (2) donor-level associations of the continuous signature with panel-excluded inflammatory pathways and AP-1-related transcription-factor (TF) activity in $CD14^+$ classical monocytes; (3) inferred AP-1/related TF architecture and target-excluded donor-level co-variation with CXCL8 and IL1B; (4) partial corroboration of AP-1-gene/CXCL8 co-expression in GSE277117 (25 baseline AS patients; no healthy controls); and (5) secondary NK-cell composition and JUN-expression findings. Panels 2, 3 and 5 summarize analyses of GSE194315 (10 AS and 29 healthy-control donors). Colors distinguish discovery/model, primary, supportive and secondary evidence. Inter-panel arrows guide the reading order; TF–ligand links represent co-variation rather than established causal regulation. The PAE–AS signature is a database-derived transcriptional construct, not a measured exposure biomarker.

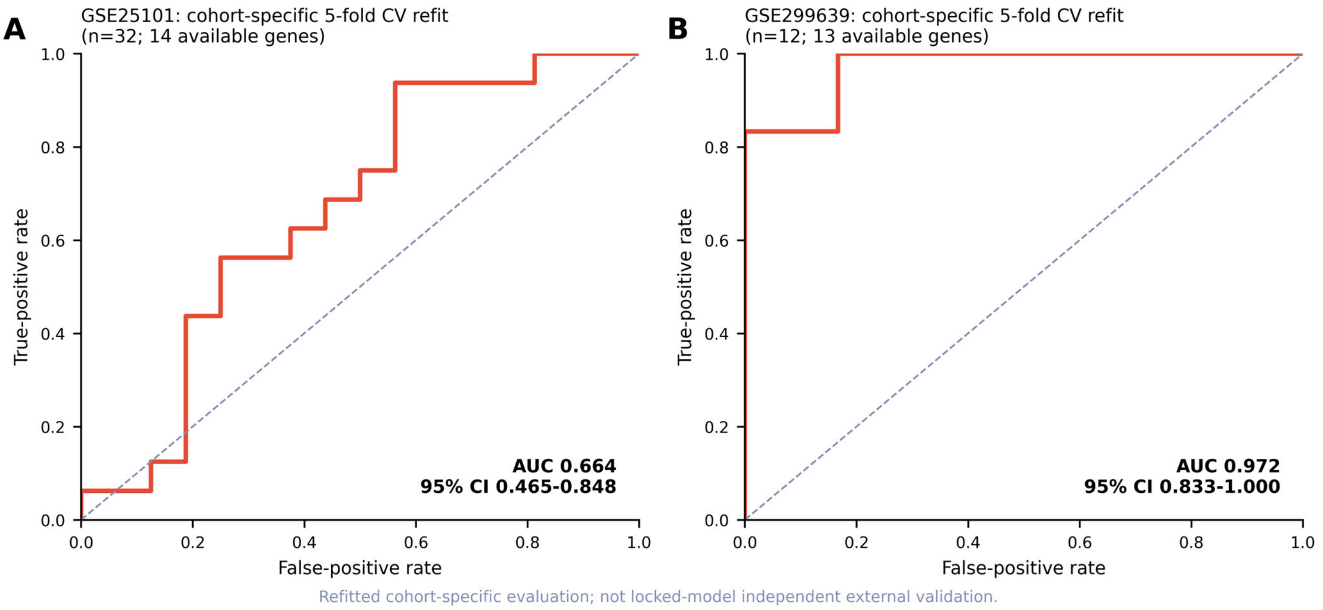

Figure S2. Secondary within-cohort cross-validated ROC estimates in two additional cohorts. Logistic-regression ROC curves using available genes from the 16-gene panel, with 5-fold cross-validation within each cohort: (A) GSE25101 (14 genes; 16 AS/16 HC; AUC = 0.664; 95% CI, 0.465–0.848) and (B) GSE299639 (13 genes; 6 AS/6 HC; AUC = 0.972; 95% CI, 0.833–1.000). Dashed line, chance. These are cohort-specific refits and are not locked-model external validation; the separate unchanged GSE73754-to-GSE25101 transfer result is reported in Results 3.2.

Supplementary Table S1. Study datasets.

Dataset	Platform / assay	Samples	Tissue	Role in this study
GSE73754	Bulk microarray	52 AS / 20 HC	Whole blood	ML candidate-gene prioritization (training)
GSE25101	Bulk microarray	16 AS / 16 HC	Whole blood	Within-cohort cross-validated evaluation; retrospective target for the locked GSE73754-trained model
GSE299639	Bulk RNA-seq	6 AS / 6 control	Peripheral blood	Small within-cohort cross-validated evaluation
GSE194315	scRNA-seq (10x)	10 AS / 29 HC (39 donors)	PBMC	Primary single-cell analyses (donor-level)
GSE277117	scRNA-seq (10x)	25 AS (baseline; no HC)	PBMC	Additional AS-only cohort; partial corroboration of monocyte AP-1-gene/CXCL8 co-variation

Supplementary Table S2. Chemical information of seven PAEs

Phthalate	Abbreviation	Chemical Formula	SMILES Structure
dimethyl phthalate	DMP	$C_{10}H_{10}O_4$	COC(=O)C1=CC=CC=C1C(=O)OC
diethyl phthalate	DEP	$C_{12}H_{14}O_4$	CCOC(=O)C1=CC=CC=C1C(=O)OCC
diisobutyl phthalate	DiBP	$C_{16}H_{22}O_4$	CC(C)COC(=O)C1=CC=CC=C1C(=O)OCC(C)C
di-n-butyl phthalate	DBP	$C_{16}H_{22}O_4$	CCCCOC(=O)C1=CC=CC=C1C(=O)OCCCC
Di(2-ethylhexyl) phthalate	DEHP	$C_{24}H_{38}O_4$	CCCCC(CC)COC(=O)C1=CC=CC=C1C(=O)OCC(CC)CCCC
diisononyl phthalate	DiNP	$C_{26}H_{42}O_4$	CC(C)CCCCCCOC(=O)C1=CC=CC=C1C(=O)OCCCCCCC(C)C
di-n-octyl phthalate	DnOP	$C_{24}H_{38}O_4$	CCCCCCCCOC(=O)C1=CC=CC=C1C(=O)OCCCCCCCC

Supplementary Table S3. Exploratory cell-pooled top DEGs, PAE–AS signature-high vs PAE–AS signature-low classical monocytes (descriptive context only)

Rank	Gene	log2FC	p-value	adj.p (BH)
1	CXCL8	4.605	<2.2e-308	<2.2e-308
2	TNF	3.102	4.62e-48	1.66e-45
3	KB-1507C5.4	3.011	3.19e-07	2.20e-05
4	IL1B	2.443	2.42e-201	1.02e-197
5	CCL4	2.339	8.09e-23	1.67e-20
6	CCL3	2.025	3.42e-124	4.12e-121
7	CXCL2	2.018	8.65e-13	1.06e-10
8	LINC00309	2	2.12e-06	1.31e-04
9	CXCR4	1.938	2.52e-117	2.66e-114
10	PPM1N	1.87	9.78e-04	3.07e-02
11	CCL4L2	1.74	5.81e-06	3.25e-04
12	ITGAL	1.618	2.56e-102	2.17e-99
13	MEFV	1.592	7.92e-93	5.35e-90
14	CTDSPL	1.551	2.28e-13	2.89e-11
15	HCAR3	1.525	3.73e-09	3.20e-07
16	COMMD7	1.514	3.13e-42	1.02e-39
17	PTGS2	1.509	8.38e-24	1.81e-21
18	TMEM258	1.489	2.34e-198	7.91e-195
19	TGFB1	1.481	6.52e-187	1.57e-183
20	STAT5B	1.459	2.05e-33	5.77e-31

Cells rather than donors were treated as independent observations in this archived exploratory analysis; the resulting P values are affected by pseudoreplication and are not used for inferential claims. Donor-level paired results are reported in Supplementary Table S4.

Supplementary Table S4. Donor-level classical-monocyte pseudobulk differential expression, PAE–AS signature-high vs PAE–AS signature-low (within-donor paired Wilcoxon, n = 39 donor tertile-pairs)

Gene	High mean (log-CPM)	Low mean (log-CPM)	Δ (High–Low)	Paired Wilcoxon P	BH q
CXCL8	10.40	7.02	+3.39	3.6×10^{-12}	1.3×10^{-11}
CXCR4	6.89	4.85	+2.04	3.6×10^{-12}	1.3×10^{-11}
TNF	5.23	2.88	+2.35	1.9×10^{-6}	2.2×10^{-6}
CCL4	5.11	3.81	+1.30	3.4×10^{-4}	3.4×10^{-4}
IL1B	8.26	7.24	+1.02	6.1×10^{-10}	1.1×10^{-9}
CCL3	8.22	7.27	+0.95	1.2×10^{-8}	1.7×10^{-8}
NFKBIA	10.70	10.23	+0.46	9.1×10^{-11}	2.1×10^{-10}

Per-donor log-CPM from summed UMI counts; paired across donors contributing both signature tertiles (≥ 10 classical monocytes each). BH q computed across the seven targeted genes; this archived tertile contrast is descriptive; the primary analysis uses a continuous score and covariate sensitivity models. CXCL8 magnitude is inflated by its inclusion in the overlap score. This direct self-inclusion is considered when interpreting the descriptive contrast.

Supplementary Table S5. Donor-level paired AP-1/immediate-early transcription-factor activity (decoupler ULM), PAE–AS signature-high vs PAE–AS signature-low classical monocytes (n = 39 donor tertile-pairs)

Transcription factor	Paired Hodges–Lehmann ΔULM (High–Low)	Paired P	BH q	Donor-bootstrap 95% CI
FOSB	+1.005	3.6×10^{-12}	7.3×10^{-12}	0.857 to 1.154
JUND	+0.947	3.6×10^{-12}	7.3×10^{-12}	0.825 to 1.064
JUN	+0.834	3.6×10^{-12}	7.3×10^{-12}	0.728 to 0.952
ATF3	+0.786	3.6×10^{-12}	7.3×10^{-12}	0.657 to 0.913
KLF6	+0.743	9.1×10^{-11}	1.0×10^{-10}	0.603 to 0.881
EGR1	+0.731	1.1×10^{-11}	1.6×10^{-11}	0.646 to 0.825
NR4A2	+0.705	7.3×10^{-12}	1.2×10^{-11}	0.594 to 0.814
FOS	+0.683	3.6×10^{-12}	7.3×10^{-12}	0.591 to 0.779
KLF4	+0.389	5.1×10^{-11}	6.4×10^{-11}	0.304 to 0.483
JUNB	+0.244	1.5×10^{-7}	1.5×10^{-7}	0.172 to 0.344

ULM activity was recomputed on donor-level profiles. Effects are paired Hodges–Lehmann estimators and confidence intervals use 5,000 donor-bootstrap resamples. This paired-tertile analysis is descriptive; primary inference uses continuous donor-level signature associations, target-excluded TF–ligand analyses and covariate sensitivity models.

Supplementary Table S6. Donor-level NK-cell pseudobulk differential expression, AS vs HC (DESeq2; 10 AS, 29 HC)

Gene	DESeq2 log_2FC (AS–HC)	DESeq2 P	DESeq2 padj
JUN	−0.995	4.77×10^{-4}	0.0497
IFNG	−0.52	0.184	0.576
KLRC1	−0.32	0.267	0.660
PRF1	+0.32	0.013	0.235
GZMB	+0.29	0.021	0.271
NKG7	+0.26	0.006	0.175
JUND	−0.26	0.239	0.634
GZMA	+0.23	0.031	0.319
JUNB	+0.21	0.491	0.810
GNLY	−0.07	0.735	0.921

Genome-wide BH correction. Among the selected NK genes, only JUN reaches significance; the cytotoxic-up/IFN-γ-down "effector dissociation" is not supported at the donor level.

Supplementary Table S7. Donor-level NK-cell composition in AS versus HC

Endpoint	AS mean (%)	HC mean (%)	AS–HC difference (percentage points; 95% CI)	P	BH q
Total NK / PBMCs	10.551	10.780	−0.230 (−4.030 to 4.011)	0.9677	0.9677
CD56bright / all NK	3.079	5.778	−2.699 (−4.575 to −0.611)	0.0332	0.0498
Conventional NK / all NK	96.341	93.306	3.035 (1.062 to 4.839)	0.0176	0.0498

Analyses included 10 AS and 29 HC donors. Values are arithmetic means of donor proportions, not pooled-cell proportions. Total NK is expressed relative to captured PBMCs; CD56bright and conventional NK are expressed relative to all NK cells. Within-NK endpoints required at least 20 NK cells per donor. Differences are AS minus HC in percentage points, with 95% confidence intervals from 5,000 donor-bootstrap resamples. P values are from ordinary least-squares regression of arcsine-square-root-transformed proportions on disease group; BH q values are corrected across the three endpoints. Conventional NK denotes the author-provided generic NK category and was not relabeled CD56dim. The all-NK denominator includes other author-annotated NK-compartment categories, so the two displayed within-NK fractions need not sum to 100%.
